# Ti_3_C_2_T_*x*_ MXene Polymer Composites for Anticorrosion: An Overview and Perspective

**DOI:** 10.1021/acsami.2c11953

**Published:** 2022-09-19

**Authors:** Ihsan Amin, Hidde van den Brekel, Kartik Nemani, Erdni Batyrev, Arnoud de Vooys, Hans van der Weijde, Babak Anasori, N. Raveendran Shiju

**Affiliations:** † Van’t Hoff Institute for Molecular Sciences, University of Amsterdam, Science Park 904, 1098 XH Amsterdam, The Netherlands; ‡ Department of Mechanical and Energy Engineering, Purdue School of Engineering and Technology and Integrated Nanosystems Development Institute, Indiana University-Purdue University Indianapolis, Indianapolis, Indiana 46202, United States; § Tata Steel Research & Development, P.O. Box 10.000, 1970CA IJmuiden, The Netherlands

**Keywords:** 2D materials, MXene, Ti_3_C_2_T*_x_*, anticorrosion, polymer composites, coatings, MAX phase

## Abstract

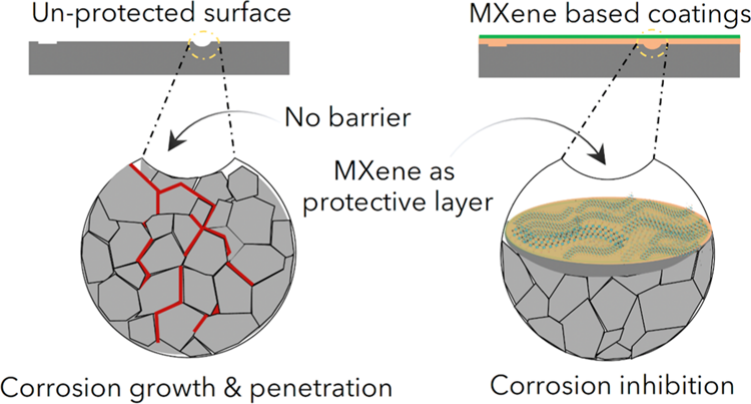

As the most studied two-dimensional (2D) material from the MXene family, Ti_3_C_2_T_*x*_ has constantly gained interest from academia and industry. Ti_3_C_2_T_*x*_ MXene has the highest electrical conductivity (up to 24,000 S cm^–1^) and one of the highest stiffness values with a Young’s modulus of ∼ 334 GPa among water-dispersible conductive 2D materials. The negative surface charge of MXene helps to disperse it well in aqueous and other polar solvents. This solubility across a wide range of solvents, excellent interface interaction, tunable surface functionality, and stability with other organic/polymeric materials combined with the layered structure of Ti_3_C_2_T_*x*_ MXene make it a promising material for anticorrosion coatings. While there are many reviews on Ti_3_C_2_T_*x*_ MXene polymer composites for catalysis, flexible electronics, and energy storage, to our knowledge, no review has been published yet on MXenes’ anticorrosion applications. In this brief report, we summarize the current progress and the development of Ti_3_C_2_T_*x*_ polymer composites for anticorrosion. We also provide an outlook and discussion on possible ways to improve the exploitation of Ti_3_C_2_T_*x*_ polymer composites as anticorrosive materials. Finally, we provide a perspective beyond Ti_3_C_2_T_*x*_ MXene composition for the development of future anticorrosion coatings.

## 1 Introduction

Ti_3_C_2_T_*x*_, the most studied two-dimensional (2D) material in the MXene family, has gained great attention since its first synthesis in 2011.^1^ The chemical formula of MXene indicates the number of atomic layers of the elements present in a sandwich-like layered morphology. For example, Ti_3_C_2_T_*x*_ consists of three layers of Ti atoms and two layers of C atoms arranged in layers of Ti–C–Ti–C–Ti. The T_*x*_ component in the formula represents the surface terminations (typically −OH, −F, −O, −Cl) existing on the outer planes of Ti as an outcome of the synthesis method.^1−3^ Thus, it is easily well-dispersed in water or other solvents, with the highest electrical conductivity (up to 24,000 S cm^–1^) and Young’s modulus (∼334 GPa) among all solution-processed 2D materials.^4−6^ In addition, the top-down synthesis method via wet chemical selective etching from its precursor, the Ti_3_AlC_2_ MAX phase,^7,8^ makes it quite scalable for industrial synthesis. Owing to these superior properties and its feasibility for solution processability, scalability, and surface functionality, various applications of Ti_3_C_2_T_*x*_ such as in catalysis,^9,10^ electromagnetic interference shielding,^11^ energy-storage applications,^12^ flexible electronics, and biosensors^13^ have been reported.

Corrosion is a tendency of a metal to convert to its oxide form. It has a significant environmental and economic impact on society. Unlike graphene, Ti_3_C_2_T_*x*_-based coatings for anticorrosion are not widely explored. For instance, Ti_3_C_2_T_*x*_ MXene was projected as a robust current collector for water desalination applications^14^ and lithium-ion batteries.^15^ Chloride anions present in saline water could corrode the current collectors beyond threshold potentials, impacting the efficiencies. The use of Ti_3_C_2_T_*x*_ MXene as a current collector is due to its high specific surface area, suitable pore structure, high redox activity, high electrical conductivity, and stability in aqueous electrolytes. These properties enable Ti_3_C_2_T_*x*_ MXene electrode operation at a high salt adsorption capacity within a large voltage window of electrochemical stability, exhibiting high reversibility without corrosion.^14^ However, despite these advantages, no works on grafting polymeric materials on Ti_3_C_2_T_*x*_ for anticorrosion protection are reported to the best of our knowledge. For anticorrosive coating applications, some reports have investigated Ti_3_C_2_T_*x*_-based polymer composites by either noncovalent or covalent functionalization^16,17^ with noncovalent functionalization, employing physical mixing of Ti_3_C_2_T_*x*_ with polymeric materials. In the first part of this review, we discuss recent research on Ti_3_C_2_T_*x*_–polymer composites for anticorrosion. Next, we present how to improve Ti_3_C_2_T_*x*_ MXene integration into polymer matrices to further enhance the anticorrosion properties of these materials. Finally, we provide a perspective beyond Ti_3_C_2_T_*x*_, which can be useful for the future development of anticorrosion coatings.

## 2 Ti_3_C_2_T_*x*_ MXene Polymer Composites for Anticorrosion

Ti_3_C_2_T_*x*_ MXene polymer composites prepared via surface functionalization will be discussed in detail in the subsequent sections. The discussion is organized as follows: (a) first, the efficacy of pristine MXenes/polymer matrix composites, followed by (b) MXene coatings derived via surface functionalization methods.

### 2.1 Pristine Ti_3_C_2_T_*x*_

**Figure 1:**
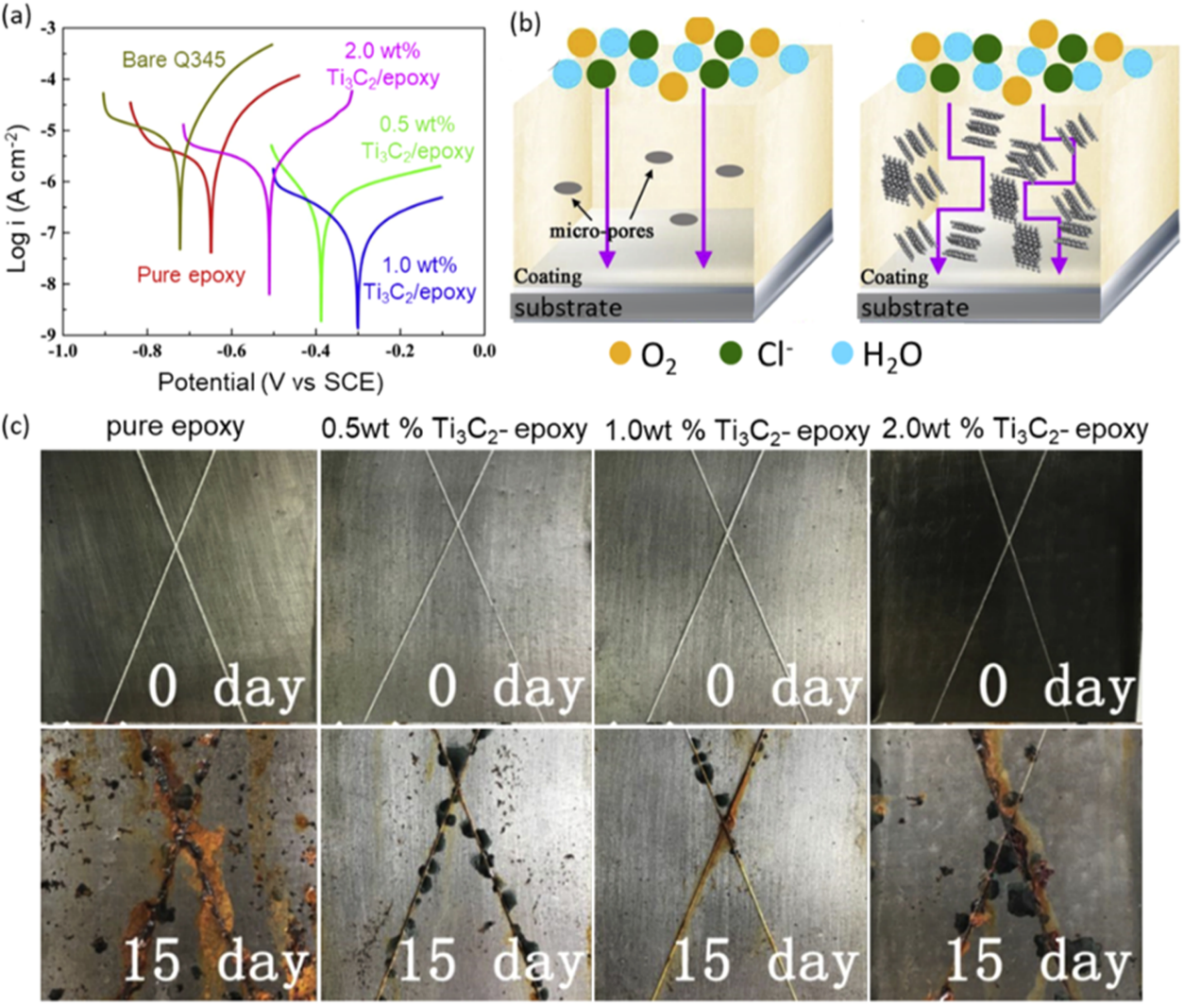
(a) Tafel plots of the anticorrosion properties of uncoated and coated samples after immersion in 3.5% NaCl for 96 h. The 1 wt %-coated sample shows the highest protection, indicated by the most positive shifting of potential value *E*_corr_ and the lowest corrosion current, *I*_corr_. Here, potential (V vs SCE) refers to potential versus saturated calomel electrode, which serves as the reference electrode. (b) Schematic illustration of corrosion process without and with a Ti_3_C_2_T_*x*_-contained epoxy coating. (c) Photographs of the samples before and after the salt spray test, where 1.0 wt % Ti_3_C_2_ offers the highest protection, in agreement with results in a. Reprinted with permission from ref (18) Copyright 2019 Elsevier.

To exploit the anticorrosion properties of pristine Ti_3_C_2_T_*x*_ nanosheets, single- to few-layer Ti_3_C_2_T_*x*_ nanosheets were physically mixed via magnetic stirring in a waterborne epoxy coating (WEC)^18,19^ or waterborne polyurethane (WPU).^20^ The anticorrosion properties of pristine Ti_3_C_2_T_*x*_ were first reported by Yan et al.,^18^ where they incorporated Ti_3_C_2_T_*x*_ nanosheets in epoxy resin with an amine curing agent. Ti_3_C_2_T_*x*_ exhibited stable dispersions in the epoxy matrix due to its hydrophilic nature, which is vital to create a perfect physical barrier for anticorrosion. Figure 1a shows the Tafel plots of the uncoated Q345 sample, pure epoxy, and Ti_3_C_2_T_*x*_/epoxy composites with different ratios of Ti_3_C_2_T_*x*_ (0.5, 1, and 2 wt % Ti_3_C_2_T_*x*_/epoxy). After immersion in 3.5% NaCl solution for 96 h, the MXene showed enhanced corrosion protection on the steel substrates compared to pure epoxy coatings. The improvement in anticorrosion properties was attributed to the presence of MXene flakes as thin film barriers for the diffusion of electrolyte and providing corrosion protection to the substrate (Figure 1b).^18^

Moreover, the 1 wt % Ti_3_C_2_T_*x*_-coated sample showed the highest protection, indicated by the shift of the potential (*E*_corr_) to the most positive value and the lowest corrosion current (*I*_corr_) as shown in Figure 1a. Table 1 shows the anticorrosion parameters of each sample, as derived from the Tafel plot in Figure 1a.

**Table 1:** Corrosion Properties for Pristine and Coated Q345 Steel Substrates after Immersion for 96 h in a 3.5% NaCl Solution^a^^b^

samples	*E*_corr_ (V)	*i*_corr_ (A·cm^–2^)	*b*_a_ (mV·dec^–1^)	*b*_c_ (mV·dec^–1^)	*R*_corr_ (MΩ·cm^2^)	*C*_R_ (mm y^–1^)	PE (%)
bare Q345	–0.75	7.51 × 10^–5^	0.120	–0.364	0.09	87.07 × 10^–3^	
pure epoxy	–0.71	1.00 × 10^–6^	0.154	–0.369	0.71	11.62 × 10^–3^	98.67
0.5 wt % Ti_3_C_2_/epoxy	–0.41	1.28 × 10^–7^	0.423	–0.094	3.20	1.48 × 10^–3^	99.83
1.0 wt % Ti_3_C_2_/epoxy	–0.29	3.39 × 10^–8^	0.257	–0.218	8.55	0.39 × 10^–3^	99.95
2.0 wt % Ti_3_C_2_/epoxy	–0.53	7.05 × 10^–7^	0.191	–0.473	0.75	8.17 × 10^–3^	99.06

a Adapted from ref (18).

b Note: *E*_corr_: corrosion potential; *I*_corr_: corrosion current density; *b*_a_: anodic Tafel slope; *b*_c_: cathodic Tafel slope; corrosion resistance, *R*_corr_ was calculated from *E*_corr_/*I*_corr_. *C*_R_: corrosion rate; PE: protection efficiency.

Figure 1c shows the optical photographs of pure epoxy and Ti_3_C_2_T_*x*_–epoxy coatings before (0 days) and after 15 days of exposure time in a salt spray chamber. In agreement with Figure 1a, 1.0 wt % Ti_3_C_2_T_*x*_–epoxy shows the highest corrosion protection, outperforming 2.0 wt % Ti_3_C_2_T_*x*_–epoxy. These results indicate that adding more Ti_3_C_2_T_*x*_ does not necessarily result in higher protection. Rather, the optimized value for the mixing of Ti_3_C_2_T_*x*_ and the epoxy where the best dispersibility can be obtained is more structurally important. The higher content of Ti_3_C_2_T_*x*_ leads to the stacking of flakes, creating higher volume densities and resulting in phase aggregation and separation between Ti_3_C_2_ and the epoxy matrix, further impeding the effectiveness of Ti_3_C_2_T_*x*_ 2D flakes as a physical barrier for anticorrosion. These results corroborate the work on graphene-based coatings, where the optimized ratio of graphene and the polymer matrix is found to be important to achieve the highest degree of protection against corrosion.^21^

Another aspect that reflects the anticorrosion properties is the impedance modulus at the lowest frequencies, |*z*|_*f*_ = 0.01 Hz, where a higher impedance modulus results in better corrosion protection. In the same study, the authors observed that for the first 2 h, the impedance moduli were 9.42 × 10^7^, 3.55 × 10^8^, 6.23 × 10^8^, and 3.95 × 10^8^ Ω cm^2^ for pure epoxy, 0.5, 1, and 2 wt % Ti_3_C_2_T_*x*_, respectively. However, after 96 h immersion in saline environments (3.5% NaCl), the impedance moduli significantly decreased to 2.27 × 10^6^, 7.6 × 10^6^, 2.96 × 10^7^, and 6.11 × 10^6^ Ω cm^2^, respectively. The decrease of the impedance modulus at longer exposures to saline environments is attributed to the instability of Ti_3_C_2_T_*x*_ due to oxidation and hydrolysis.^22−24^ It is known that despite its superior intrinsic properties, Ti_3_C_2_T_*x*_ flakes are prone to hydrolysis and oxidation in hydrated environments and potential transformation to TiO_*x*_ and TiO_2_.^17,23−25^ The degradation is usually initiated at the defect and edge sites of MXenes and is a multistep process where the defect sites undergo systematic hydrolysis with the formation of TiO_2_ as the final degraded product.^26^ This chemical degradation may impede the anticorrosion behavior of MXene in the long term, therefore requiring strategies to mitigate oxidation.

The oxidation of MXenes due to the reaction with dissolved oxygen can be limited by storing them in solutions saturated with inert gas.^24^ Another way to slow the oxidation rates of MXenes is by freezing at ultralow temperatures (−20 or −80 °C), extending the shelf life to two years.^27^ Stability of MXene can be effectively improved by hydrogen annealing but causes loss of dispersibility in solvents and surface reactivity.^28^ When excess aluminum was used in the synthesis mixture of Ti_3_AlC_2_ resulting Ti_3_C_2_T_*x*_ MXene had lower number of Ti and C vacancies and exhibited a better oxidation resistance.^29^ A long-term storage of MXenes in an aqueous solution utilizing hydration chemistry with nontoxic inorganic salts inhibits the attack of MXene by free water and oxygen molecules.^30−32^ As a result, oxidation can be largely inhibited, prolonging the shelf life to up to 400 days with negligible loss of surface chemistry. Other methods such as surface functionalization^33,34^ with organic ligands can maintain MXene chemical stability for long-term applications such as anticorrosion additives.

### 2.2 Surface-Functionalized Ti_3_C_2_T_*x*_ MXene

Aminopropyl triethoxysilane (APTES) is the most used silane for surface functionalization. The primary amino functional groups in APTES offer several possibilities for postfunctionalization from bioconjugation to nanoparticle impregnation. Ji et al.^35^ reported that functionalized Ti_3_C_2_T_*x*_ with APTES demonstrates improved stability against oxidation with adjustable hydrophilicity in comparison to pristine Ti_3_C_2_T_*x*_ MXene.

**Figure 2:**
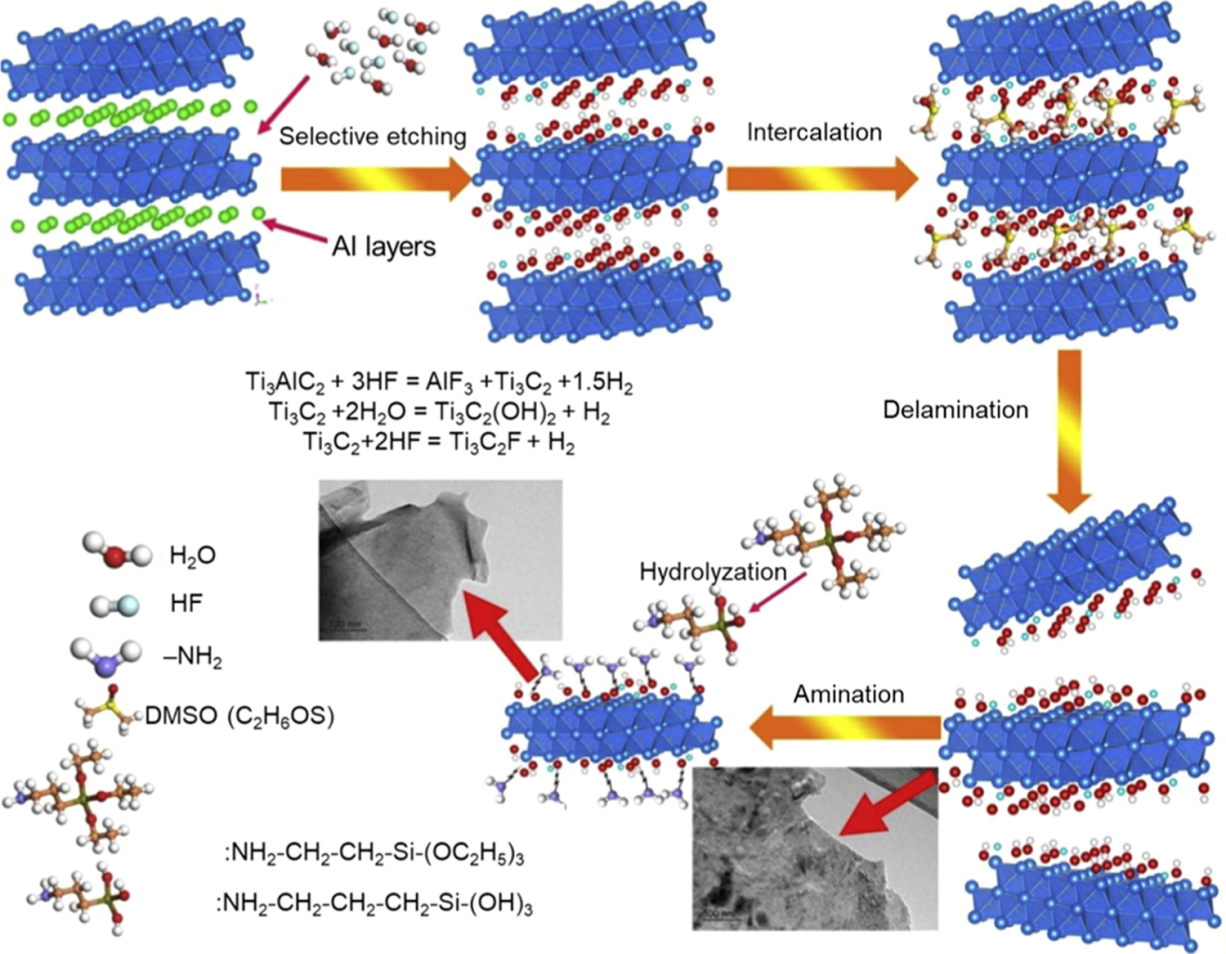
Schematic illustration of synthesis and surface functionalization of Ti_3_C_2_T_*x*_ with APTES. Reprinted with permission from ref (36) Copyright 2020 Elsevier.

Yan et al.^36^ were the first to investigate the anticorrosion properties of amino-functionalized Ti_3_C_2_T_*x*_ MXene. In their work, APTES was first attached to Ti_3_C_2_T_*x*_ via a simple wet deposition, using the abundant hydroxyl functional groups on Ti_3_C_2_T_*x*_ in the colloidal solution form. Figure 2 shows the schematic illustration of the preparation of Ti_3_C_2_T_*x*_ MXene and its surface functionalization with APTES. The amino-functionalized Ti_3_C_2_T_*x*_ exhibited higher mechanical properties and showed better dispersibility in water, in comparison to pristine Ti_3_C_2_T_*x*_.^36^ Note that the synthesis of Ti_3_C_2_T_*x*_ MXene has been reported in numerous studies and reviews elsewhere^3,37−40^ and is not in the scope of this review.

**Figure 3:**
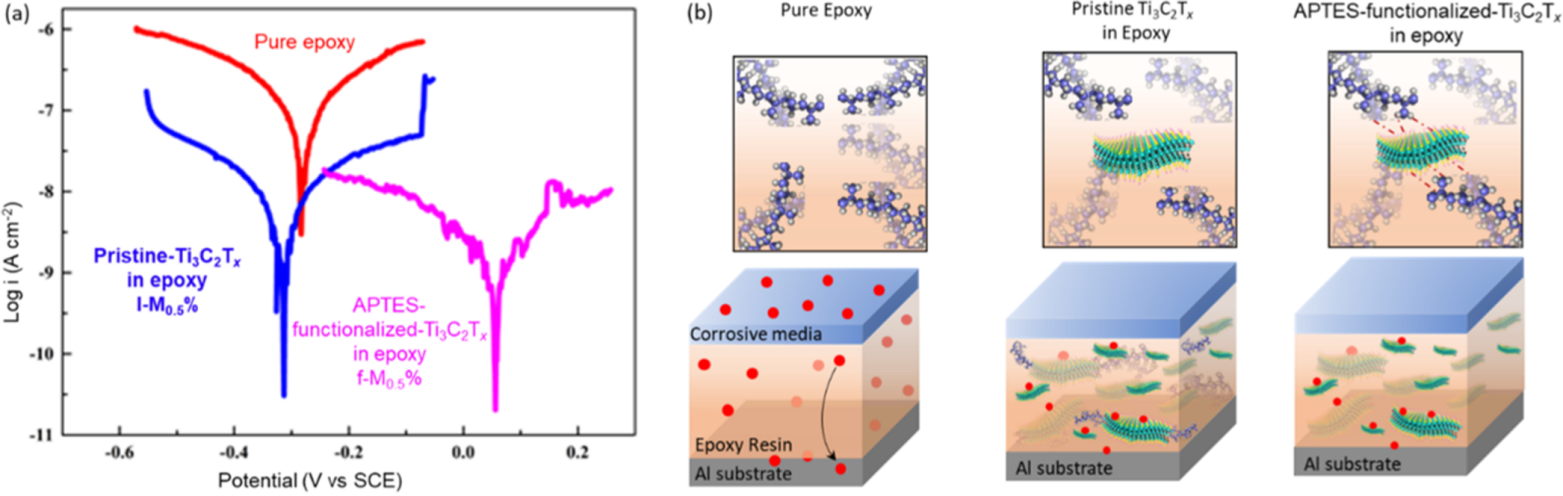
(a) Tafel plot of pure epoxy, l-M_0.5%_, and f-M_0.5%_ after 4-week immersion in a 3.5% NaCl solution. f-M_0.5%_ shows the best anticorrosion performance, indicated by the most positive value. The potential (V vs SCE) refers to potential versus saturated calomel electrode, which serves as the reference electrode. (b) Schematic illustration of the corrosion protection process in pure epoxy, pristine Ti_3_C_2_T_*x*_/epoxy, and APTES-functionalized/epoxy coatings. Reprinted with permission from ref (36) Copyright 2020 Elsevier.

Composites with 0.25 and 0.5 wt % of pristine Ti_3_C_2_T_*x*_ (0.25 l-M and 0.5 l-M, respectively) and 0.25 and 0.5 wt % APTES-functionalized Ti_3_C_2_T_*x*_ (0.25 and 0.5 f-M, respectively) in the waterborne epoxy polymer were prepared by physical mixing. The addition of pristine Ti_3_C_2_T_*x*_ and APTES-functionalized Ti_3_C_2_T_*x*_ has enhanced the anticorrosion properties of the epoxy coatings (Figure 3a) where MXene 2D flakes acted as a physical barrier to the corrosion agents (Figure 3b). The corrosion resistance was 2.34 × 10^8^ Ω cm^2^ for pure epoxy. The functionalized Ti_3_C_2_T_*x*_ exhibits higher corrosion resistance in comparison to pristine Ti_3_C_2_T_*x*_. The 0.5 wt % APTES-functionalized Ti_3_C_2_T_*x*_/epoxy coatings (f-M_0.5%_) demonstrated the best anticorrosion protection, as indicated by the most positive value in the Tafel plot (Figure 3a and Table 2). f-M_0.5%_ showed the highest corrosion protection of 3.09 × 10^9^ Ω cm^2^. However, after 4 weeks of immersion in 3.5% NaCl, a significant decrease in corrosion resistance was observed for all samples. The pure epoxy exhibited the highest degradation to 3.45 × 10^5^ Ω cm^2^, whereas the 0.5 wt % APTES-functionalized Ti_3_C_2_T_*x*_/epoxy coatings exhibited the lowest degradation (from 3.09 × 10^9^ to 1.02 × 10^7^ Ω cm^2^). The study shows the importance of the ligand functionalization of Ti_3_C_2_T_*x*_ in improving its dispersibility as well as maintaining its chemical stability while slowing down degradation due to oxidation in the polymer matrix.^36^

**Table 2:** Anticorrosion Resistance of the Pure Epoxy, Pristine Ti_3_C_2_T*_x_*/Epoxy (l-M), and APTES-Functionalized Ti_3_C_2_T*_x_*/Epoxy (f-M)-Coated Samples, after 1-Day and 4-Week Immersion in 3.5% NaCl^a^

sample	*R*_corr_ (1 day) (Ω cm^2^)	*R*_corr_ (4 weeks) (Ω cm^2^)
pure	2.34 × 10^8^	3.46 × 10^5^
0.25 l-M	6.45 × 10^8^	4.68 × 10^6^
0.5 l-M	7.94 × 10^8^	5.89 × 10^6^
0.25 f-M	2.04 × 10^9^	8.91 × 10^6^
0.5 f-M	3.09 × 10^9^	1.02 × 10^7^

a Adapted from ref (36).

**Figure 4:**
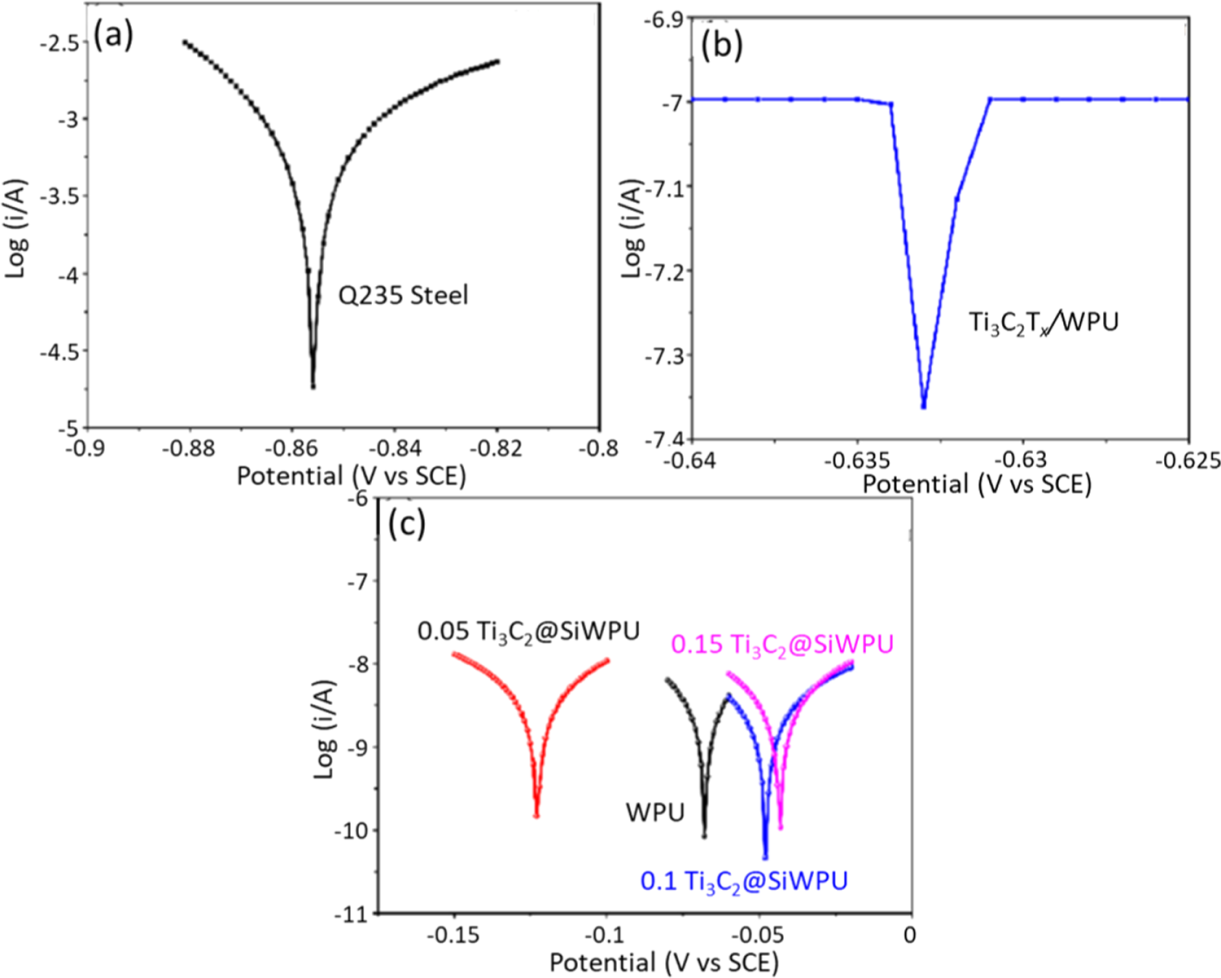
Tafel plots of (a) uncoated Q235 steel, (b) pristine Ti_3_C_2_/WPU, and (c) WPU and functionalized Ti_3_C_2_@Si/WPU. The functionalized coated samples exhibit a positive shift value, indicating the highest corrosion protection. The potential (V vs SCE) refers to potential versus saturated calomel electrode, which serves as the reference electrode. Reprinted with permission from ref (41) Copyright 2021 Elsevier.

Similarly, Zhang et al.,^41^ reported surface functionalization of [3-(2-aminoethyl) aminopropyl] trimethoxysilane (AEAPTES) on Ti_3_C_2_T_*x*_ MXene. The AEAPTES-functionalized Ti_3_C_2_T_*x*_ (named Ti_3_C_2_@Si) was then mixed with waterborne polyurethane (WPU) with Ti_3_C_2_T_*x*_ ratios of 0.05, 0.1, and 0.15 wt %. Figure 4 shows the Tafel plots for the uncoated steel substrate, pristine Ti_3_C_2_T_*x*_/WPU, and WPU and Ti_3_C_2_@Si. Ti_3_C_2_@Si exhibited a positive shift in the Tafel plot (Figure 4c). The 0.1 wt % Ti_3_C_2_T_*x*_ sample showed the lowest current density, indicating the best anticorrosion performance, outperforming 0.15, 0.05 wt %, WPU, Ti_3_C_2_T_*x*_ /WPU, and bare Q235 steel substrate.

The parameters extracted from the Tafel plots (Table 3) confirm better corrosion protection of the functionalized-Ti_3_C_2_T_*x*_ coated samples in comparison to pristine Ti_3_C_2_T_*x*_ MXene. The results indicated that pristine Ti_3_C_2_T_*x*_ MXene in WPU was less effective in anticorrosion compared with pure WPU (Table 3). The poor performance of pristine Ti_3_C_2_T_*x*_ MXene in WPU may be attributed to the intermittent and noncontinuous interfacial adhesion between pristine Ti_3_C_2_T_*x*_ and WPU. This may create spaces or micropores that facilitate the corrosive ions to permeate and subsequently propagate the degradation beneath the coating. All functionalized Ti_3_C_2_T_*x*_ MXene–WPU coatings showed higher corrosion protection with 0.1 wt % exhibiting the lowest current density of 2.67 × 10^–9^ A cm^–2^ and the highest contact resistance of 3.05 × 10^6^ Ω·cm^2^. Impressively, after 42 days of immersion in 3.5% NaCl, no degradation was observed. This finding corroborates with the work of Yan et al.,^36^ where the surface functionalization of Ti_3_C_2_T_*x*_ with APTES was found to enhance the chemical enhance and stabilize the corrosion resistance.

**Table 3:** Resistance Parameters as Derived from the Tafel Plot in Figure 4, Adapted from ref (41)

sample	*E*_corr_ (V)	*I*_corr_ (A cm^–2^)	*R*_p_ (Ω)	*B*_a_	*B*_c_	CR (mm year^–1^)
Q235 steel	–0.86	3.23 × 10^–4^	11 × 10^0^	2.26	1.58	3.76 × 10^0^
WPU	–0.07	2.32 × 10^–8^	1.9 × 10^6^	4.90	4.98	2.74 × 10^–4^
Ti_3_C_2_/WPU	–0.633	2.8 × 10^–5^	6.63 × 10^4^	0.08	0.15	3.27 × 10^–1^
0.05% Ti_3_C_2_@Si/WPU	–0.12	9.94 × 10^–9^	2.09 × 10^6^	10.78	10.18	1.16 × 10^–4^
0.1% Ti_3_C_2_@Si/WPU	–0.05	2.67 × 10^–9^	3.05 × 10^6^	26.94	26.46	3.11 × 10^–5^
0.15% Ti_3_C_2_@Si/WPU	–0.04	3.48 × 10^–9^	2.19 × 10^6^	30.02	27.11	4.06 × 10^–5^

**Figure 5:**
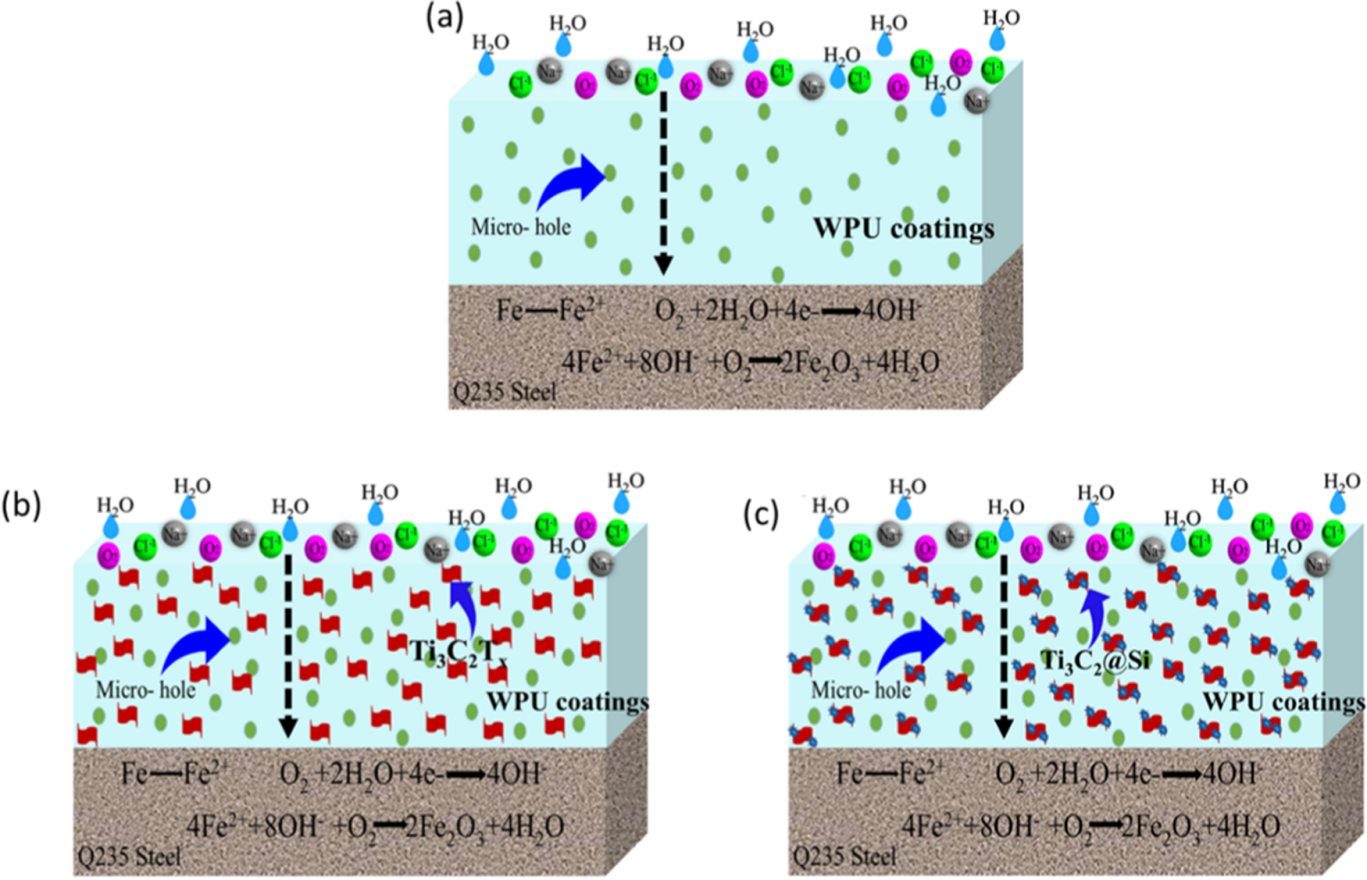
Mechanism of the corrosion protection for (a) pristine WPU, (b) Ti_3_C_2_T_*x*_/WPU, and (c) functionalized Ti_3_C_2_T_*x*_@Si/WPU. Reprinted with permission from ref (41) copyright 2021 Elsevier.

Figure 5 shows the mechanism of the corrosion protection of pure WPU, Ti_3_C_2_T_*x*_/WPU, and functionalized Ti_3_C_2_@Si/WPU. Steels coated with pure WPU corrode easily, probably due to the defects and micron-sized holes through which the ions can permeate with relative ease. Contrary to expectations, Ti_3_C_2_/WPU coatings were less efficient than pristine WPU. This may be due to abundant oxygen functional groups on Ti_3_C_2_T_*x*_ surfaces, attracting water molecules and other corrosive media, acting as initiator sites for oxidation. The introduction of amino functional groups on Ti_3_C_2_T_*x*_ facilitates intercalation with isocyanate groups in WPU, giving a strong and compact structure and leading to stable dispersions of Ti_3_C_2_@Si in WPU. Therefore, a network of an effective barrier was formed by good compatibility and dispersibility while creating complex diffusion paths, thereby slowing the diffusion rates. Importantly, the functionalized Ti_3_C_2_@Si increased the hydrophobicity of WPU, which decreased the absorption of water and enhanced the corrosion performance of Ti_3_C_2_/WPU composite coatings. These findings are similar to those of graphene-based materials,^21,42,43^ where covalently functionalized graphene demonstrated better corrosion protection compared to pristine graphene.

### 2.3 Ti_3_C_2_T_*x*_/PANI Composites

**Figure 6:**
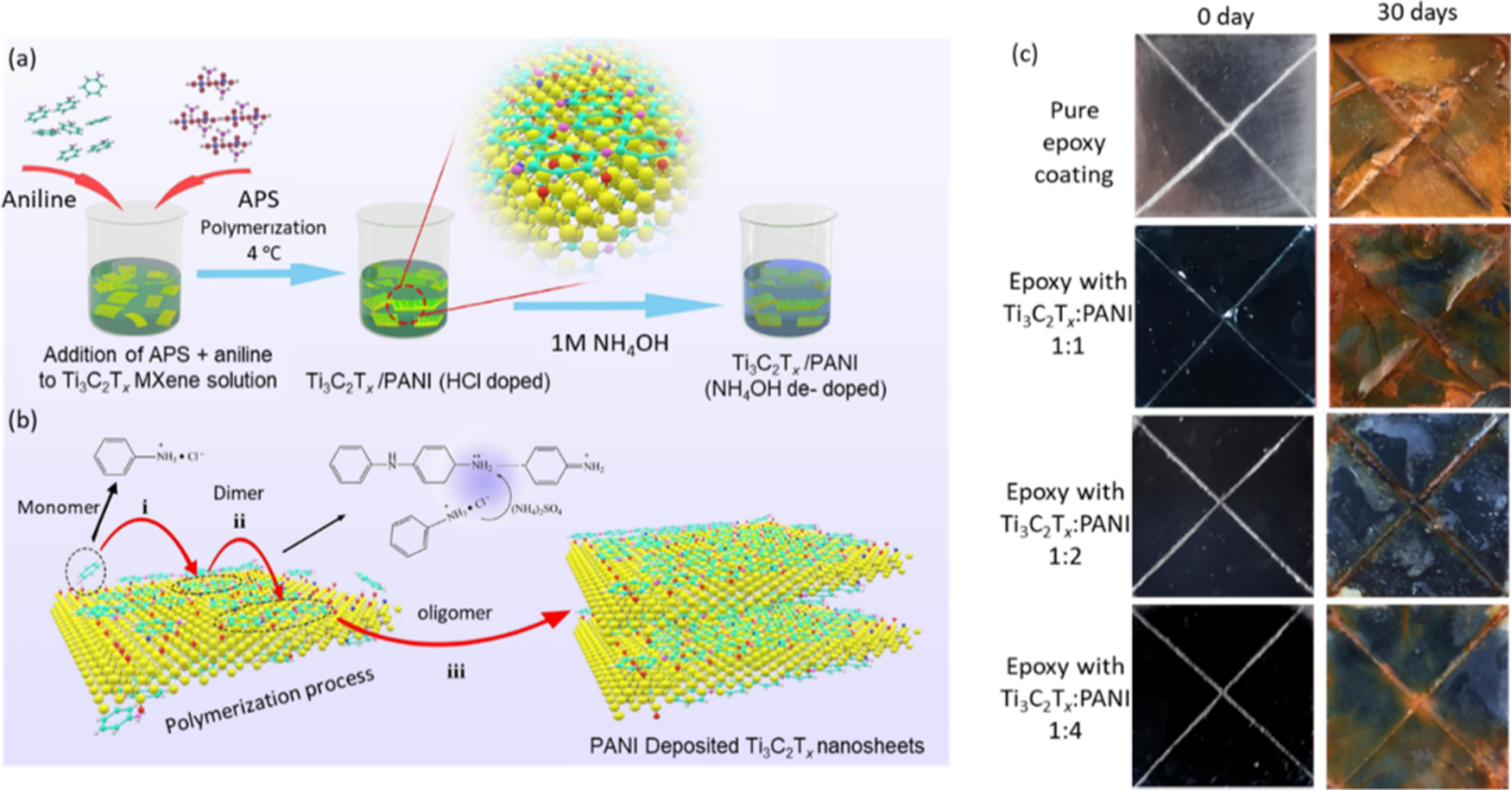
Schematic Illustration of the preparation of Ti_3_C_2_/PANI composites. (a) Preparation of Ti_3_C_2_ nanosheets. (b) Synthesis of Ti_3_C_2_/PANI composites (TPCs) with the mechanism of oxidative polymerization of aniline on Ti_3_C_2_. (c) Photograph of the samples before and after the salt spray test for 30 days. Reprinted with permission from ref (51) copyright 2021 Elsevier.

Conductive polymers (CPs) such as polythiophene (PT),^44^ polypyrrole (PPy),^45,46^ and polyaniline (PANI)^47−49^ exhibit anticorrosion properties. Among these, PANI has attracted more attention for anticorrosion owing to its ease of synthesis, thermal stability, and reversible acid/base doping/dedoping.^50^ Cai et al.^51^ combined PANI and the 2D Ti_3_C_2_T_*x*_ to enhance the anticorrosion properties of waterborne epoxy (WEP) resins. Ti_3_C_2_T_*x*_/PANI composites were prepared via in situ polymerization (Figure 6a,b) in Ti_3_C_2_T_*x*_/PANI mass ratios of 1:1, 1:2, and 1:4, followed by sandwiching the Ti_3_C_2_T_*x*_/PANI composite between two WEP layers to create multilayer coatings on mild steel substrates.

Among all of the Ti_3_C_2_T_*x*_/PANI composite coatings, Ti_3_C_2_T_*x*_/PANI with a 1:2 ratio exhibited the best corrosion protection (Figure 6c). The initial impedance modulus after 1-day immersion in 3.5% NaCl was 7 × 10^8^ Ω·cm^2^ and decreased to 1 order of magnitude to 1.05 × 10^7^ Ω·cm^2^ after 5 weeks of immersion. Furthermore, Ti_3_C_2_T_*x*_/PANI (1:2) sprayed with salts for 45 days demonstrated minimum contents of oxygen and chlorine. The excellent corrosion protection was attributed to the 3D structures of Ti_3_C_2_T_*x*_/PANI composites, which served as a reservoir and as a trap for corrosive ions.

### 2.4 Ti_3_C_2_T_*x*_/Graphene Hybrid Composites

Graphene has excellent impermeable and physical barrier properties. However, its ability to be dispersed in the polymer matrix is poor. To improve graphene dispersion, the covalent functionalization of graphene with polymeric materials is an option. Another novel strategy is to combine graphene and Ti_3_C_2_T_*x*_ to achieve graphene/Ti_3_C_2_T_*x*_ heterostructures. Since Ti_3_C_2_T_*x*_ is hydrophilic, the resulting graphene/Ti_3_C_2_T_*x*_ heterostructures are also hydrophilic and may show beneficial synergistic effects for anticorrosion.

**Figure 7:**
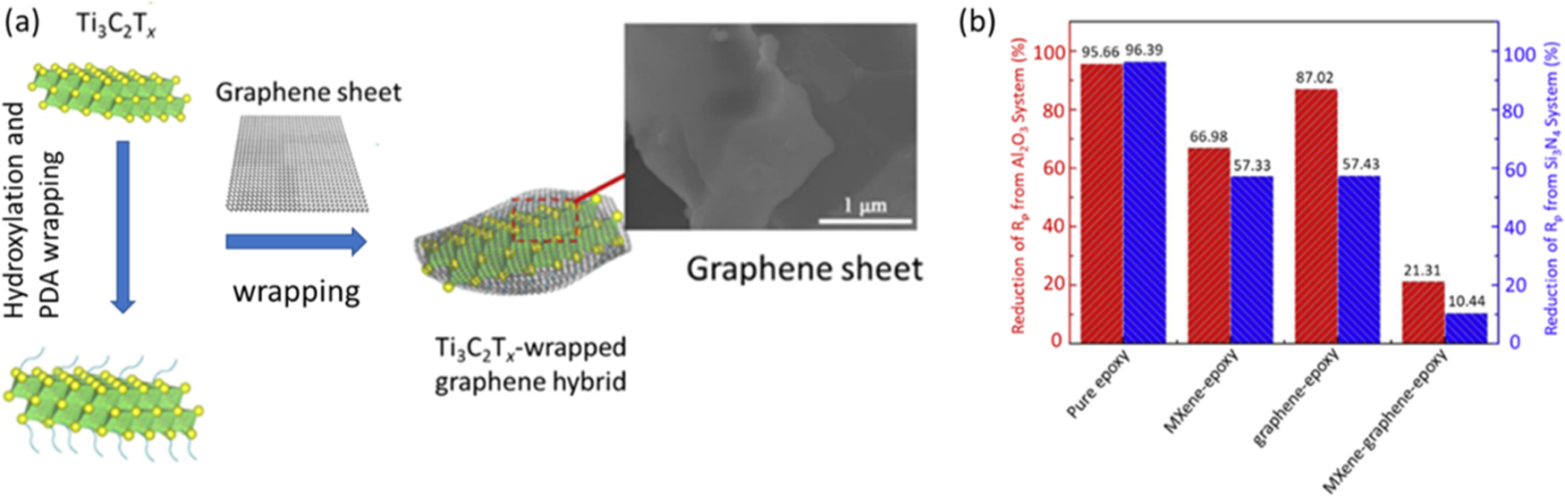
(a) Schematic illustration of the synthesis of Ti_3_C_2_T_*x*_/graphene heterostructures with wrapping structures where MXene sheets are wrapped by graphene sheets. (b) Reduction of the corrosion polarization resistance *R*_p_ for all coatings. MG-EP shows the lowest reduction, while pure EP exhibits the highest loss of corrosion resistance. Reprinted with permission from ref (52) copyright 2020 Elsevier.

Recently, Yan et al.,^52^ have demonstrated the anticorrosion properties of Ti_3_C_2_T_*x*_/graphene heterostructures in which Ti_3_C_2_T_*x*_ is wrapped by graphene. First, they prepared the Ti_3_C_2_T_*x*_/graphene heterostructures by making a graphene-wrapped Ti_3_C_2_T_*x*_ via polydopamine interfacial chemistry (Figure 7a). Ti_3_C_2_T_*x*_/graphene was then mixed with the epoxy to make Ti_3_C_2_T_*x*_/graphene–epoxy coating composites. The corrosion properties of the Ti_3_C_2_T_*x*_/graphene–epoxy coating composites in 3.5% NaCl were studied.

The Ti_3_C_2_T_*x*_/graphene–epoxy coating exhibited a corrosion resistance modulus of 2.14 × 10^9^ Ω·cm^2^, higher than those of pure epoxy (1.0^6^ × 10^8^ Ω·cm^2^), Ti_3_C_2_T_*x*_ MXene–epoxy (1.51 × 10^9^ Ω·cm^2^), and graphene–epoxy (1.53 × 10^9^ Ω·cm^2^). The Ti_3_C_2_T_*x*_/graphene–epoxy coating exhibited a significant decrease in corrosion impedance modulus of 21.3 and 10.4% after the wear test with Al_2_O_3_ and Si_3_N_4_ balls, respectively. For pure epoxy, the corrosion impedance modulus was decreased by 95.7 and 96.4% after the wear test with Al_2_O_3_ and Si_3_N_4_, respectively (Figure 7b). The enhanced performance of the Ti_3_C_2_T_*x*_/graphene–epoxy composite was attributed to the (i) thermal conductivity and excellent lubricant properties of Ti_3_C_2_T_*x*_ and graphene, (ii) dual hybrid surfaces that form protective films, and (iii) the synergistic effects of Ti_3_C_2_T_*x*_/graphene-interweaved structures that greatly improved the anticorrosion properties of organic coatings.

## 3 Conclusions and Future Perspectives

We have discussed the current progress on the Ti_3_C_2_T_*x*_ MXene for anticorrosion applications. Both pristine and functionalized Ti_3_C_2_T_*x*_ may be used for making anticorrosive coatings. Physical mixing of Ti_3_C_2_T_*x*_ with waterborne epoxy (WEP) or polyurethane (PU) is the most popular route for preparing anticorrosion coatings. In general, studies on the behavior of the Ti_3_C_2_T_*x*_ MXene in corrosive environments indicate that its hydrophilicity has both advantages and disadvantages. Ti_3_C_2_T_*x*_ hydrophilicity improves its dispersibility in WEP and PU matrices. However, the abundant oxygen functional groups on the Ti_3_C_2_T_*x*_ surface may trigger more corrosion. Ti_3_C_2_T_*x*_ MXene is prone to chemical degradation due to active hydrolysis and surface oxidation, leading to the formation of titanium oxide,^24^ which may impede the long-term corrosion protection.

There are at least three main approaches to slow down or prevent the oxidation and chemical degradation of MXenes and to improve their shelf life. The first route is to improve the purity and stoichiometry of the precursor MAX phase to enhance the quality of the resulting MXene flakes by lowering the number of defects in the resulting MXene 2D flakes.^29^ The second approach is to increase the flake size and decrease the concentrations of the defects during the selective etching and delamination processes.^53^ The third method is to improve MXenes’ oxidation resistance by decorating their surface with organic/inorganic moieties.^31,33,54^ The presence of multiple species of ions in-between the MXene sheets can also influence the rate of oxidation.

Surface functionalization of Ti_3_C_2_T_*x*_ is important to maintain its chemical stability for long-term application in anticorrosion coatings.^36,41^ The attachment of organic functional groups was used to functionalize Ti_3_C_2_T_*x*_ to maintain its chemical stability.^35^ A strategy that may further improve the chemical stability of Ti_3_C_2_T_*x*_ is the covalent functionalization with polymer brushes.^55^ However, the grafting of polymer brushes on Ti_3_C_2_T_*x*_ for its application in anticorrosion is not explored yet. This can be an easier task in comparison to the assembly of MXene with other 2D materials, such as graphene or hexagonal boron nitride. The abundant functional groups on the surface of Ti_3_C_2_T_*x*_ can be functionalized with initiators to perform surface-initiated atom transfer radical polymerization. Furthermore, direct photografting, known as self-initiated photografting and photopolymerization (SIPGP), can also be used, which is a one-step polymerization process where no initiator attachment is needed. Polymer brush grafting methods via SIPGP have been utilized on other 2D materials such as graphene,^56,57^ graphitic carbon nitride,^58^ and hexagonal boron nitride.^59^ Unlike graphene,^60^ Ti_3_C_2_T_*x*_-based coatings for anticorrosion are not widely explored and the research area of MXenes for anticorrosion coatings is at its initial stage. Ti_3_C_2_T_*x*_ is only one composition in the large compositional space of MXenes, which suggests the potential of this field due to the tailorable compositions and properties of MXenes.

## 4 Beyond Ti_3_C_2_T_x_ MXene

Research on the anticorrosive behavior of this large family of 2D layered carbides/nitrides and carbonitrides is very limited and is yet to expand beyond Ti_3_C_2_T_*x*_ MXene. MXenes consisting of transition metals with high electronegativities may be more effective for inhibiting the propagation of corrosion. The introduction of two different MXene compositions in aqueous or other solvent media together can improve their combined efficacy for inhibiting corrosion. For example, the use of different MXene types mixed together has shown improved electrochemical activity.^61^ Preparation of MXenes in nonaqueous, polar solvents can further eliminate the potential of oxidation due to hydrolysis. In addition, a more in-depth understanding of surface terminations' effect toward high impedance behavior and stability in saline environments must be developed, which leads to lower protection/inhibition of corrosion.

Since corrosion is a surface phenomenon, tailoring of surface functional groups is vital to develop coatings that are resistant to oxide growth, therefore requiring more oxidation-resistant species on the basal (outer) planes of MXenes. MXenes with two transition metals, known as double transition metal MXenes, as either random solid solutions or in-plane ordered or out-of-plane ordered MXenes^62−64^ can potentially inhibit corrosion via the contribution from the transition metals order/disorder, as well as via providing better control on their charge transport properties.^65^

Extending the concept of multiple M elements in MXenes, the newest addition of entropy-stabilized MAX phases and the isolation of high-entropy MXenes with multiple principal metals have further expanded the scope of MXenes for exploration toward anticorrosion materials.^66−69^ These systems, where the metal elements have random occupancies, exhibit a high rate of disorder with diverse electronegativities. This can potentially contribute to lower rates of oxidation and extend the service life of coatings due to the availability of nonhomogeneous active sites for corrosion initiation. The diversity of MXenes in terms of constituent elements, layer thicknesses with two to five layers of transition metals, and different surface terminations^70^ provide a platform to further expand the available tools in corrosion science and engineering.

## References

[ref1] Naguib M.; Kurtoglu M.; Presser V.; Lu J.; Niu J.; Heon M.; Hultman L.; Gogotsi Y.; Barsoum M. W. Two-Dimensional Nanocrystals Produced by Exfoliation of Ti3AlC2. Adv. Mater. 2011, 23, 4248–4253. 10.1002/adma.201102306. 21861270

[ref2] Slot T. K.; Riley N.; Shiju N. R.; Medlin J. W.; Rothenberg G. An Experimental Approach for Controlling Confinement Effects at Catalyst Interfaces. Chem. Sci. 2020, 11, 11024–11029. 10.1039/D0SC04118A. 34123192 PMC8162257

[ref3] Fu Z.; Wang N.; Legut D.; Si C.; Zhang Q.; Du S.; Germann T. C.; Francisco J. S.; Zhang R. Rational Design of Flexible Two-Dimensional MXenes with Multiple Functionalities. Chem. Rev. 2019, 119, 11980–12031. 10.1021/acs.chemrev.9b00348. 31710485

[ref4] Zhang J.; Kong N.; Uzun S.; Levitt A.; Seyedin S.; Lynch P. A.; Qin S.; Han M.; Yang W.; Liu J.; Wang X.; Gogotsi Y.; Razal J. M. Scalable Manufacturing of Free-Standing, Strong Ti3C2Tx MXene Films with Outstanding Conductivity. Adv. Mater. 2020, 32, 2001093 10.1002/adma.202070393. 32309891

[ref5] Lipatov A.; Lu H.; Alhabeb M.; Anasori B.; Gruverman A.; Gogotsi Y.; Sinitskii A. Elastic Properties of 2D Ti3C2Tx MXene Monolayers and Bilayers. Sci. Adv. 2018, 4, eaat0491 10.1126/sciadv.aat0491. 29922719 PMC6003751

[ref6] Shayesteh Zeraati A.; Mirkhani S. A.; Sun P.; Naguib M.; Braun P. V.; Sundararaj U. Improved Synthesis of Ti3C2Tx MXenes Resulting in Exceptional Electrical Conductivity, High Synthesis Yield, and Enhanced Capacitance. Nanoscale 2021, 13, 3572–3580. 10.1039/D0NR06671K. 33538284

[ref7] Barsoum M. W. The MN+1AXN phases: A New Class of Solids: Thermodynamically Stable Nanolaminates. Prog. Solid State Chem. 2000, 28, 201–281. 10.1016/S0079-6786(00)00006-6.

[ref8] Tzenov N. V.; Barsoum M. W. Synthesis and Characterization of Ti3AlC2. J. Am. Ceram. Soc. 2004, 83, 825–832. 10.1111/j.1151-2916.2000.tb01281.x.

[ref9] Slot T. K.; Natu V.; Ramos-Fernandez E. V.; Sepúlveda-Escribano A.; Barsoum M.; Rothenberg G.; Shiju N. R. Enhancing Catalytic Epoxide Ring-Opening Selectivity Using Surface-Modified Ti3C2Tx MXenes. 2D Mater. 2021, 8, 035003 10.1088/2053-1583/abe951.

[ref10] Slot T. K.; Yue F.; Xu H.; Ramos-Fernandez E. V.; Sepúlveda-Escribano A.; Sofer Z.; Rothenberg G.; Shiju N. R. Surface Oxidation of Ti3C2Tx Enhances the Catalytic Activity of Supported Platinum Nanoparticles in Ammonia Borane Hydrolysis. 2D Mater. 2021, 8, 015001 10.1088/2053-1583/ababef.

[ref11] Han M.; Shuck C. E.; Rakhmanov R.; Parchment D.; Anasori B.; Koo C. M.; Friedman G.; Gogotsi Y. Beyond Ti3C2Tx: MXenes for Electromagnetic Interference Shielding. ACS Nano 2020, 14, 5008–5016. 10.1021/acsnano.0c01312. 32163265

[ref12] Xu X. D.; Zhang Y. L.; Sun H. Y.; Zhou J. W.; Yang F.; Li H.; Chen H.; Chen Y. C.; Liu Z.; Qiu Z. P.; Wang D.; Ma L. P.; Wang J. W.; Zeng Q. G.; Peng Z. Q. Progress and Perspective: MXene and MXene-Based Nanomaterials for High-Performance Energy Storage Devices. Adv. Electron. Mater. 2021, 7, 2000967 10.1002/aelm.202000967.

[ref13] Huang W.; Hu L.; Tang Y.; Xie Z.; Zhang H. Recent Advances in Functional 2D MXene-Based Nanostructures for Next-Generation Devices. Adv. Funct. Mater. 2020, 30, 2005223 10.1002/adfm.202005223.

[ref14] Buczek S.; Barsoum M. L.; Uzun S.; Kurra N.; Andris R.; Pomerantseva E.; Mahmoud K. A.; Gogotsi Y. Rational Design of Titanium Carbide MXene Electrode Architectures for Hybrid Capacitive Deionization. Energy Environ. Mater. 2020, 3, 398–404. 10.1002/eem2.12110.

[ref15] Wang C.-H.; Kurra N.; Alhabeb M.; Chang J.-K.; Alshareef H. N.; Gogotsi Y. Titanium Carbide (MXene) as a Current Collector for Lithium-Ion Batteries. ACS Omega 2018, 3, 12489–12494. 10.1021/acsomega.8b02032. 31457980 PMC6644544

[ref16] Carey M.; Barsoum M. W. MXene Polymer Nanocomposites: a Review. Mater. Today Adv. 2021, 9, 100120 10.1016/j.mtadv.2020.100120.

[ref17] Riazi H.; Nemani S. K.; Grady M. C.; Anasori B.; Soroush M. Ti3C2 MXene–Polymer Nanocomposites and their Applications. J. Mater. Chem. A 2021, 9, 8051–8098. 10.1039/D0TA08023C.

[ref18] Yan H.; Li W.; Li H.; Fan X.; Zhu M. Ti3C2 MXene Nanosheets Toward High-Performance Corrosion Inhibitor for Epoxy Coating. Prog. Org. Coat. 2019, 135, 156–167. 10.1016/j.porgcoat.2019.06.013.

[ref19] Yan H.; Cai M.; Wang J.; Zhang L.; Li H.; Li W.; Fan X.; Zhu M. Insight into Anticorrosion/Antiwear Behavior of Inorganic-Organic Multilayer Protection System Composed of Nitriding Layer and Epoxy Coating with Ti3C2Tx MXene. Appl. Surf. Sci. 2021, 536, 147974 10.1016/j.apsusc.2020.147974.

[ref20] Sheng X.; Li S.; Huang H.; Zhao Y.; Chen Y.; Zhang L.; Xie D. Anticorrosive and UV-Blocking Waterborne Polyurethane Composite Coating Containing Novel Two-Dimensional Ti3C2 MXene Nanosheets. J. Mater. Sci. 2021, 56, 4212–4224. 10.1007/s10853-020-05525-2.

[ref21] Zhang F.; Liu W.; Liang L.; Wang S.; Shi H.; Xie Y.; Yang M.; Pi K. The Effect of Functional Graphene Oxide Nanoparticles on Corrosion Resistance of Waterborne Polyurethane. Colloids Surf., A 2020, 591, 124565 10.1016/j.colsurfa.2020.124565.

[ref22] Iqbal A.; Hong J.; Ko T. Y.; Koo C. M. Improving Oxidation Stability of 2D MXenes: Synthesis, Storage Media, and Conditions. Nano Convergence 2021, 8, 9 10.1186/s40580-021-00259-6. 33723803 PMC7960843

[ref23] Habib T.; Zhao X.; Shah S. A.; Chen Y.; Sun W.; An H.; Lutkenhaus J. L.; Radovic M.; Green M. J. Oxidation Stability of Ti3C2Tx MXene Nanosheets in Solvents and Composite Films. npj 2D Mater. Appl. 2019, 3, 8 10.1038/s41699-019-0089-3.

[ref24] Zhang C. J.; Pinilla S.; McEvoy N.; Cullen C. P.; Anasori B.; Long E.; Park S.-H.; Seral-Ascaso A.; Shmeliov A.; Krishnan D.; Morant C.; Liu X.; Duesberg G. S.; Gogotsi Y.; Nicolosi V. Oxidation Stability of Colloidal Two-Dimensional Titanium Carbides (MXenes). Chem. Mater. 2017, 29, 4848–4856. 10.1021/acs.chemmater.7b00745.

[ref25] Cao F.; Zhang Y.; Wang H.; Khan K.; Tareen A. K.; Qian W.; Zhang H.; Ågren H. Recent Advances in Oxidation Stable Chemistry of 2D MXenes. Adv. Mater. 2022, 34, 2107554 10.1002/adma.202107554. 34816509

[ref26] Huang S.; Mochalin V. N. Hydrolysis of 2D Transition-Metal Carbides (MXenes) in Colloidal Solutions. Inorg. Chem. 2019, 58, 1958–1966. 10.1021/acs.inorgchem.8b02890. 30649863

[ref27] Zhang J.; Kong N.; Hegh D.; Usman K. A. S.; Guan G.; Qin S.; Jurewicz I.; Yang W.; Razal J. M. Freezing Titanium Carbide Aqueous Dispersions for Ultra-long-term Storage. ACS Appl. Mater. Interfaces 2020, 12, 34032–34040. 10.1021/acsami.0c06728. 32615749

[ref28] Lee Y.; Kim S. J.; Kim Y.-J.; Lim Y.; Chae Y.; Lee B.-J.; Kim Y.-T.; Han H.; Gogotsi Y.; Ahn C. W. Oxidation-Resistant Titanium Carbide MXene Films. J. Mater. Chem. A 2020, 8, 573–581. 10.1039/C9TA07036B.

[ref29] Mathis T. S.; Maleski K.; Goad A.; Sarycheva A.; Anayee M.; Foucher A. C.; Hantanasirisakul K.; Shuck C. E.; Stach E. A.; Gogotsi Y. Modified MAX Phase Synthesis for Environmentally Stable and Highly Conductive Ti3C2 MXene. ACS Nano 2021, 15, 6420–6429. 10.1021/acsnano.0c08357. 33848136

[ref30] Wang X.; Wang Z.; Qiu J. Stabilizing MXene by Hydration Chemistry in Aqueous Solution. Angew. Chem., Int. Ed. 2021, 60, 26587–26591. 10.1002/anie.202113981. 34729881

[ref31] VahidMohammadi A.; Mojtabavi M.; Caffrey N. M.; Wanunu M.; Beidaghi M. Assembling 2D MXenes into Highly Stable Pseudocapacitive Electrodes with High Power and Energy Densities. Adv. Mater. 2019, 31, 1806931 10.1002/adma.201806931. 30589131

[ref32] Matthews K.; Zhang T.; Shuck C. E.; VahidMohammadi A.; Gogotsi Y. Guidelines for Synthesis and Processing of Chemically Stable Two-Dimensional V2CTx MXene. Chem. Mater. 2022, 34, 499–509. 10.1021/acs.chemmater.1c03508.

[ref33] Natu V.; Hart J. L.; Sokol M.; Chiang H.; Taheri M. L.; Barsoum M. W. Edge Capping of 2D-MXene Sheets with Polyanionic Salts To Mitigate Oxidation in Aqueous Colloidal Suspensions. Angew. Chem., Int. Ed. 2019, 58, 12655–12660. 10.1002/anie.201906138. 31293049

[ref34] Choi E.; Lee J.; Kim Y.-J.; Kim H.; Kim M.; Hong J.; Kang Y. C.; Koo C. M.; Kim D. W.; Kim S. J. Enhanced Stability of Ti3C2Tx MXene Enabled by Continuous ZIF-8 Coating. Carbon 2022, 191, 593–599. 10.1016/j.carbon.2022.02.036.

[ref35] Ji J.; Zhao L.; Shen Y.; Liu S.; Zhang Y. Covalent Stabilization and Functionalization of MXene via Silylation Reactions with Improved Surface Properties. FlatChem 2019, 17, 100128 10.1016/j.flatc.2019.100128.

[ref36] Yan H.; Cai M.; Li W.; Fan X.; Zhu M. Amino-functionalized Ti3C2Tx with Anti-Corrosive/Wear Function for Waterborne Epoxy Coating. J. Mater. Sci. Technol. 2020, 54, 144–159. 10.1016/j.jmst.2020.05.002.

[ref37] Gao L.; Li C.; Huang W.; Mei S.; Lin H.; Ou Q.; Zhang Y.; Guo J.; Zhang F.; Xu S.; Zhang H. MXene/Polymer Membranes: Synthesis, Properties, and Emerging Applications. Chem. Mater. 2020, 32, 1703–1747. 10.1021/acs.chemmater.9b04408.

[ref38] Jimmy J.; Kandasubramanian B. Mxene Functionalized Polymer Composites: Synthesis and Applications. Eur. Polym. J. 2020, 122, 109367 10.1016/j.eurpolymj.2019.109367.

[ref39] Chen X.; Zhao Y.; Li L.; Wang Y.; Wang J.; Xiong J.; Du S.; Zhang P.; Shi X.; Yu J. MXene/Polymer Nanocomposites: Preparation, Properties, and Applications. Polym. Rev. 2021, 61, 80–115. 10.1080/15583724.2020.1729179.

[ref40] Alhabeb M.; Maleski K.; Anasori B.; Lelyukh P.; Clark L.; Sin S.; Gogotsi Y. Guidelines for Synthesis and Processing of Two-Dimensional Titanium Carbide (Ti3C2Tx MXene). Chem. Mater. 2017, 29, 7633–7644. 10.1021/acs.chemmater.7b02847.

[ref41] Zhang F.; Liu W.; Wang S.; Liu C.; Shi H.; Liang L.; Pi K. Surface Functionalization of Ti3C2Tx and its Application in Aqueous Polymer Nanocomposites for Reinforcing Corrosion Protection. Composites, Part B 2021, 217, 108900 10.1016/j.compositesb.2021.108900.

[ref42] Wang H.; Qin S.; Yang X.; Fei G.; Tian M.; Shao Y.; Zhu K.; Waterborne A. Uniform Graphene-Poly(urethane-acrylate) Complex with Enhanced Anticorrosive Properties Enabled by Ionic Interaction. Chem. Eng. J. 2018, 351, 939–951. 10.1016/j.cej.2018.06.151.

[ref43] Wen J.-G.; Geng W.; Geng H.-Z.; Zhao H.; Jing L.-C.; Yuan X.-T.; Tian Y.; Wang T.; Ning Y.-J.; Wu L. Improvement of Corrosion Resistance of Waterborne Polyurethane Coatings by Covalent and Noncovalent Grafted Graphene Oxide Nanosheets. ACS Omega 2019, 4, 20265–20274. 10.1021/acsomega.9b02687. 31815229 PMC6893952

[ref44] Bahrani M.; Sharif M.; Amirazodi K. Preparation and Characterization of Polythiophene/Graphene Oxide/Epoxy Nanocomposite Coatings with Advanced Properties. Polym. Bull. 2022, 79, 263–284. 10.1007/s00289-020-03529-1.

[ref45] Contri G.; Zimmermann C. A.; Ramoa S. D. A. D. S.; Schmitz D. P.; Ecco L. G.; Barra G. M. O.; Fedel M. Polypyrrole Modified E-Coat Paint for Corrosion Protection of Aluminum AA1200. Front. Mater. 2020, 7, 1–9. 10.3389/fmats.2020.00045.

[ref46] Lu F.; Liu C.; Chen Z.; Veerabagu U.; Chen Z.; Liu M.; Hu L.; Xia H.; Cha L.; Zhang W. Polypyrrole-Functionalized Boron Nitride Nanosheets for High-Performance Anti-Corrosion Composite Coating. Surf. Coat. Technol. 2021, 420, 127273 10.1016/j.surfcoat.2021.127273.

[ref47] Li A.; Chen S.; Ma Z.; Sun M.; Zhu G.; Zhang Y.; Wang W. Corrosion Protection Properties of Polyvinyl Butyral/Polyaniline-Graphene Oxide/Poly(methylhydrosiloxane) Composite Coating for AA2024 aluminum alloy. Diamond Relat. Mater. 2021, 116, 108397 10.1016/j.diamond.2021.108397.

[ref48] Kadri Y.; Srasra E.; Bekri-Abbes I.; Herrasti P. Facile and Eco-friendly Synthesis of Polyaniline/ZnO Composites for Corrosion Protection of AA-2024 Aluminium Alloy. J. Electroanal. Chem. 2021, 893, 115335 10.1016/j.jelechem.2021.115335.

[ref49] Gao M.; Quan X.; Wang J.; Wang Z. Preparation and Characterization of Coatings Incorporated with Poly(aniline-co-nitroaniline) Nanoparticles Having Antifouling and Anticorrosion Behavior. Ind. Eng. Chem. Res. 2020, 59, 22173–22186. 10.1021/acs.iecr.0c05329.

[ref50] Gao F.; Mu J.; Bi Z.; Wang S.; Li Z. Recent Advances of Polyaniline Composites in Anticorrosive Coatings: A Review. Prog. Org. Coat. 2021, 151, 106071 10.1016/j.porgcoat.2020.106071.

[ref51] Cai M.; Yan H.; Li Y.; Li W.; Li H.; Fan X.; Zhu M. Ti3C2Tx/PANI Composites with Tunable Conductivity Towards Anticorrosion Application. Chem. Eng. J. 2021, 410, 128310 10.1016/j.cej.2020.128310.

[ref52] Yan H.; Zhang L.; Li H.; Fan X.; Zhu M. Towards High-Performance Additive of Ti3C2/Graphene Hybrid with a Novel Wrapping Structure in Epoxy Coating. Carbon 2020, 157, 217–233. 10.1016/j.carbon.2019.10.034.

[ref53] Maleski K.; Ren C. E.; Zhao M.-Q.; Anasori B.; Gogotsi Y. Size-Dependent Physical and Electrochemical Properties of Two-Dimensional MXene Flakes. ACS Appl. Mater. Interfaces 2018, 10, 24491–24498. 10.1021/acsami.8b04662. 29956920

[ref54] Zhao X.; Vashisth A.; Prehn E.; Sun W.; Shah S. A.; Habib T.; Chen Y.; Tan Z.; Lutkenhaus J. L.; Radovic M.; Green M. J. Antioxidants Unlock Shelf-Stable Ti3C2Tx (MXene) Nanosheet Dispersions. Matter 2019, 1, 513–526. 10.1016/j.matt.2019.05.020.

[ref55] Lee J. T.; Wyatt B. C.; Davis G. A.; Masterson A. N.; Pagan A. L.; Shah A.; Anasori B.; Sardar R. Covalent Surface Modification of Ti3C2Tx MXene with Chemically Active Polymeric Ligands Producing Highly Conductive and Ordered Microstructure Films. ACS Nano 2021, 15, 19600–19612. 10.1021/acsnano.1c06670. 34786933

[ref56] Steenackers M.; Gigler A. M.; Zhang N.; Deubel F.; Seifert M.; Hess L. H.; Lim C. H. Y. X.; Loh K. P.; Garrido J. A.; Jordan R.; Stutzmann M.; Sharp I. D. Polymer Brushes on Graphene. J. Am. Chem. Soc. 2011, 133, 10490–10498. 10.1021/ja201052q. 21639111

[ref57] Zhang T.; Rodriguez R. D.; Amin I.; Gasiorowski J.; Rahaman M.; Sheng W.; Kalbacova J.; Sheremet E.; Zahn D. R. T.; Jordan R. Bottom-Up Fabrication of Graphene-Based Conductive Polymer Carpets for Optoelectronics. J. Mater. Chem. C 2018, 6, 4919–4927. 10.1039/C8TC00554K.

[ref58] Sheng W.; Li W.; Tan D.; Zhang P.; Zhang E.; Sheremet E.; Schmidt B. V. K. J.; Feng X.; Rodriguez R. D.; Jordan R.; Amin I. Polymer Brushes on Graphitic Carbon Nitride for Patterning and as a SERS Active Sensing Layer via Incorporated Nanoparticles. ACS Appl. Mater. Interfaces 2020, 12, 9797–9805. 10.1021/acsami.9b21984. 31999093 PMC7050013

[ref59] Sheng W.; Amin I.; Neumann C.; Dong R.; Zhang T.; Wegener E.; Chen W.-L.; Förster P.; Tran H. Q.; Löffler M.; Winter A.; Rodriguez R. D.; Zschech E.; Ober C. K.; Feng X.; Turchanin A.; Jordan R. Polymer Brushes on Hexagonal Boron Nitride. Small 2019, 15, 1805228 10.1002/smll.201805228. 30932320

[ref60] Amin I.; Batyrev E.; de Vooys A.; van der Weijde H.; Shiju N. R. Covalent Polymer Functionalization of Graphene/Graphene Oxide and its Application as Anticorrosion Materials. 2D Mater. 2022, 9, 032002 10.1088/2053-1583/ac54ee.

[ref61] VahidMohammadi A.; Liang W.; Mojtabavi M.; Wanunu M.; Beidaghi M. 2D Titanium and Vanadium Carbide MXene Heterostructures for Electrochemical Energy Storage. Energy Storage Mater. 2021, 41, 554–562. 10.1016/j.ensm.2021.06.014.

[ref62] Anasori B.; Xie Y.; Beidaghi M.; Lu J.; Hosler B. C.; Hultman L.; Kent P. R. C.; Gogotsi Y.; Barsoum M. W. Two-Dimensional, Ordered, Double Transition Metals Carbides (MXenes). ACS Nano 2015, 9, 9507–9516. 10.1021/acsnano.5b03591. 26208121

[ref63] Hong W.; Wyatt B. C.; Nemani S. K.; Anasori B. Double Transition-Metal MXenes: Atomistic Design of Two-Dimensional Carbides and Nitrides. MRS Bull. 2020, 45, 850–861. 10.1557/mrs.2020.251.

[ref64] Rosen J.; Dahlqvist M.; Tao Q.; Hultman L. In- and Out-of-Plane Ordered MAX Phases and Their MXene Derivatives. In 2D Metal Carbides and Nitrides (MXenes): Structure, Properties and Applications, Anasori B.; Gogotsi Y., Eds. Springer: Cham, 2019; Vol. 1, pp 37–52.

[ref65] Anasori B.; Shi C.; Moon E. J.; Xie Y.; Voigt C. A.; Kent P. R. C.; May S. J.; Billinge S. J. L.; Barsoum M. W.; Gogotsi Y. Control of Electronic Properties of 2D Carbides (MXenes) by Manipulating their Transition Metal Layers. Nanoscale Horiz. 2016, 1, 227–234. 10.1039/C5NH00125K. 32260625

[ref66] Nemani S. K.; Zhang B.; Wyatt B. C.; Hood Z. D.; Manna S.; Khaledialidusti R.; Hong W.; Sternberg M. G.; Sankaranarayanan S. K. R. S.; Anasori B. High-Entropy 2D Carbide MXenes: TiVNbMoC3 and TiVCrMoC3. ACS Nano 2021, 15, 12815–12825. 10.1021/acsnano.1c02775. 34128649

[ref67] Du Z.; Wu C.; Chen Y.; Cao Z.; Hu R.; Zhang Y.; Gu J.; Cui Y.; Chen H.; Shi Y.; Shang J.; Li B.; Yang S. High-Entropy Atomic Layers of Transition-Metal Carbides (MXenes). Adv. Mater. 2021, 33, 2101473 10.1002/adma.202101473. 34365658

[ref68] Du Z.; Wu C.; Chen Y.; Zhu Q.; Cui Y.; Wang H.; Zhang Y.; Chen X.; Shang J.; Li B.; Chen W.; Liu C.; Yang S.; High-Entropy Carbonitride M. A. X. Phases and Their Derivative MXenes. Adv. Energy Mater. 2022, 12, 2103228 10.1002/aenm.202103228.

[ref69] Etman A. S.; Zhou J.; Rosen J. Ti1.1V0.7CrxNb1.0Ta0.6C3Tz high-entropy MXene freestanding films for charge storage applications. Electrochem. Commun. 2022, 137, 107264 10.1016/j.elecom.2022.107264.

[ref70] VahidMohammadi A.; Rosen J.; Gogotsi Y. The world of Two-Dimensional Carbides and Nitrides (MXenes). Science 2021, 372, eabf1581 10.1126/science.abf1581. 34112665

